# Supplementary material for: Clarithromycin Versus Metronidazole in First-Line Helicobacter Pylori Triple Eradication Therapy Based on Resistance to Antimicrobial Agents: Meta-Analysis
Source: J Clin Med. 2020 Feb 17;9(2):543. doi: 10.3390/jcm9020543 (PMC7073899; doi:10.3390/jcm9020543)
Supplement: Supplementary file 1 [file jcm-09-00543-s001.pdf]

Journal of
Clinical Medicine

MDPI

A. Intention-to-treat analysis

Study or Subgroup	PAM Events	PAM Total	PAC Events	PAC Total	Weight	Risk Ratio M-H, Fixed, 95% CI	Year
Lind 1996	30	124	35	234	4.0%	1.62 [1.05, 2.50]	1996
Labenz 1996	5	33	1	31	0.2%	4.70 [0.58, 37.99]	1996
Miyaji 1997	10	70	15	74	2.4%	0.70 [0.34, 1.46]	1997
Pieramico 1997	12	61	8	58	1.3%	1.43 [0.63, 3.24]	1997
Misiewicz 1997	44	131	17	121	2.9%	2.39 [1.45, 3.95]	1997
Stack 1998	2	19	1	19	0.2%	2.00 [0.20, 20.24]	1998
Harris 1998	23	64	9	62	1.5%	2.48 [1.25, 4.92]	1998
Pilotto (2) 1999	10	50	9	50	1.5%	1.11 [0.49, 2.50]	1999
Pilotto (1) 1999	8	38	6	40	1.0%	1.40 [0.54, 3.67]	1999
Savarino 1999	18	80	34	80	5.5%	0.53 [0.33, 0.86]	1999
Uygun 1999	57	134	35	88	6.9%	1.07 [0.77, 1.48]	1999
Huang 2000	9	30	4	37	0.6%	2.77 [0.95, 8.13]	2000
Malfertheiner 2003	67	167	56	169	9.1%	1.21 [0.91, 1.61]	2003
Namiot 2004	14	45	14	45	2.3%	1.00 [0.54, 1.85]	2004
Bhatia 2004	48	70	41	76	6.4%	1.27 [0.98, 1.65]	2004
Koivisto 2005	23	106	10	110	1.6%	2.39 [1.19, 4.77]	2005
Sun 2005	8	45	8	58	1.1%	1.29 [0.52, 3.17]	2005
Sancar 2006	5	10	4	24	0.4%	3.00 [1.01, 8.91]	2006
Namiot 2008	30	82	19	84	3.1%	1.62 [0.99, 2.63]	2008
Filipec 2009	60	297	50	295	8.2%	1.19 [0.85, 1.67]	2009
Loghmari 2012	20	39	14	46	2.1%	1.68 [0.99, 2.87]	2012
Lahbabi 2013	31	104	25	115	3.9%	1.37 [0.87, 2.16]	2013
Nishizawa 2015	6	64	16	60	2.7%	0.35 [0.15, 0.84]	2015
Lee 2015	53	170	61	170	9.9%	0.87 [0.64, 1.17]	2015
Gungor 2015	48	100	58	100	9.5%	0.83 [0.64, 1.08]	2015
Adachi 2017	6	72	20	68	3.4%	0.28 [0.12, 0.66]	2017
Mabe 2018	6	169	48	137	8.6%	0.10 [0.04, 0.23]	2018
**Total (95% CI)**		**2374**		**2451**	**100.0%**	**1.07 [0.98, 1.17]**	
Total events	653		618				

Heterogeneity: $Chi^2 = 105.81$, df = 26 ($P < 0.00001$); $I^2 = 75\%$
Test for overall effect: Z = 1.49 (P = 0.14)

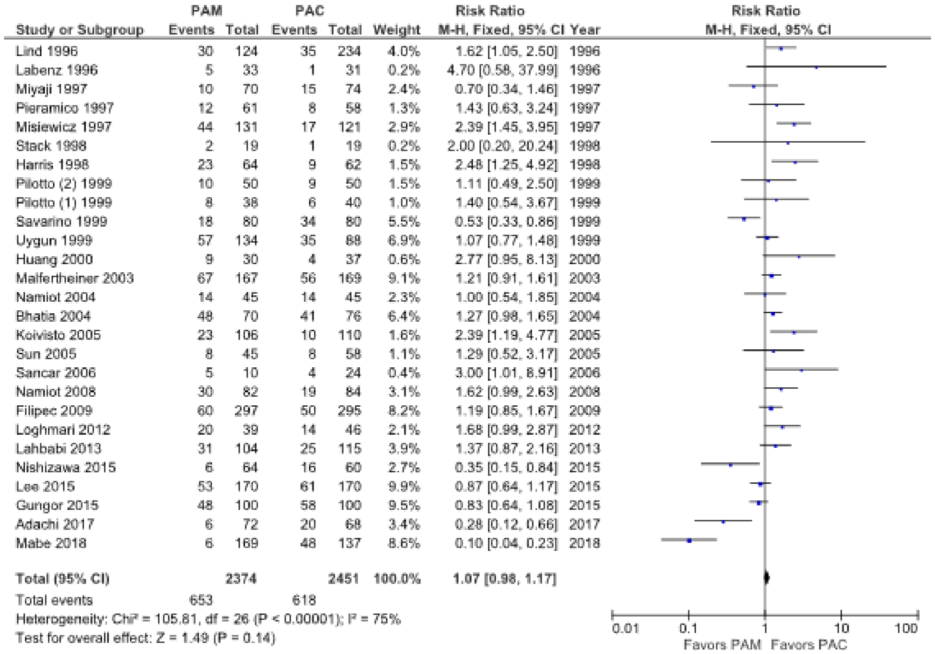

B. Per-protocol analysis

Study or Subgroup	PAM Events	PAM Total	PAC Events	PAC Total	Weight	Risk Ratio M-H, Fixed, 95% CI	Year
Labenz 1996	5	33	1	31	0.2%	4.70 [0.58, 37.99]	1996
Lind 1996	21	114	18	209	3.0%	2.14 [1.19, 3.85]	1996
Misiewicz 1997	30	113	11	114	2.6%	2.75 [1.45, 5.22]	1997
Pieramico 1997	8	57	4	54	1.0%	1.89 [0.61, 5.93]	1997
Miyaji 1997	10	70	15	74	3.5%	0.70 [0.34, 1.46]	1997
Harris 1998	12	50	6	59	1.3%	2.36 [0.96, 5.83]	1998
Stack 1998	2	17	0	18	0.1%	5.28 [0.27, 102.58]	1998
Pilotto (1) 1999	5	35	6	40	1.3%	0.95 [0.32, 2.85]	1999
Savarino 1999	8	70	23	69	5.5%	0.34 [0.16, 0.71]	1999
Pilotto (2) 1999	6	46	5	46	1.2%	1.20 [0.39, 3.66]	1999
Uygun 1999	44	121	26	79	7.5%	1.10 [0.75, 1.64]	1999
Huang 2000	6	27	2	35	0.4%	3.89 [0.85, 17.77]	2000
Malfertheiner 2003	32	132	14	127	3.4%	2.20 [1.23, 3.92]	2003
Bhatia 2004	30	52	19	54	4.4%	1.64 [1.07, 2.52]	2004
Namiot 2004	9	40	7	38	1.7%	1.22 [0.51, 2.95]	2004
Koivisto 2005	23	106	10	110	2.3%	2.39 [1.19, 4.77]	2005
Sun 2005	7	44	6	56	1.3%	1.48 [0.54, 4.10]	2005
Sancar 2006	5	10	4	24	0.6%	3.00 [1.01, 8.91]	2006
Namiot 2008	26	78	16	81	3.7%	1.69 [0.98, 2.89]	2008
Filipec 2009	45	282	33	278	7.9%	1.34 [0.89, 2.04]	2009
Loghmari 2012	20	39	14	46	3.1%	1.68 [0.99, 2.87]	2012
Lahbabi 2013	30	103	23	113	5.2%	1.43 [0.89, 2.30]	2013
Nishizawa 2015	4	62	13	57	3.2%	0.28 [0.10, 0.82]	2015
Gungor 2015	44	96	45	87	11.2%	0.89 [0.66, 1.19]	2015
Lee 2015	22	139	34	143	8.0%	0.67 [0.41, 1.08]	2015
Adachi 2017	4	70	18	66	4.4%	0.21 [0.07, 0.59]	2017
Mabe 2018	5	162	46	134	12.0%	0.09 [0.04, 0.22]	2018
**Total (95% CI)**		**2168**		**2242**	**100.0%**	**1.11 [0.99, 1.24]**	
Total events	463		419				

Heterogeneity: $Chi^2 = 109.74$, df = 26 ($P < 0.00001$); $I^2 = 76\%$
Test for overall effect: Z = 1.75 (P = 0.08)

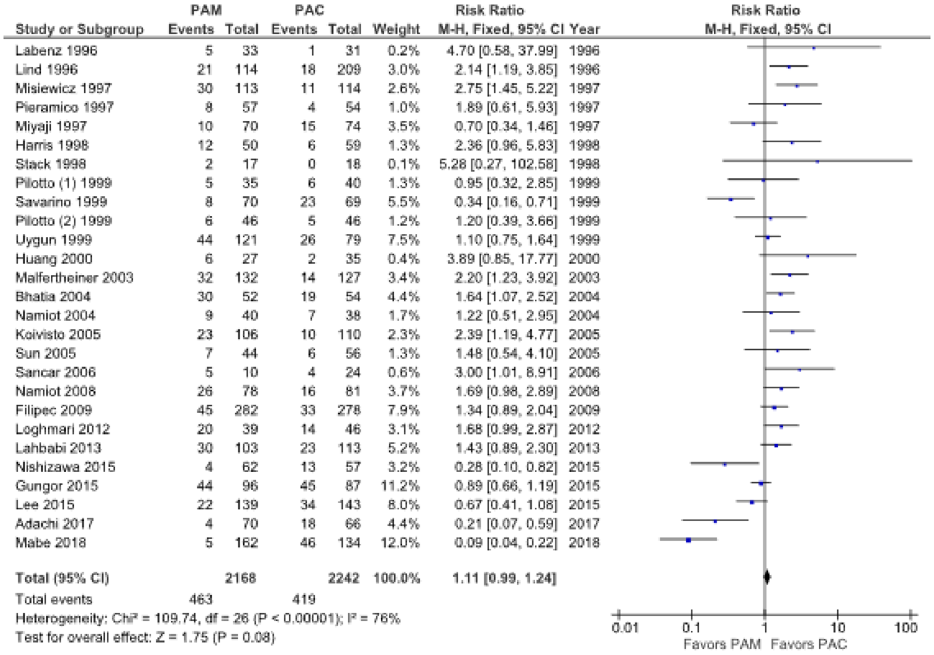

Figure S1. Forest plots of overall eradication rates between PAM therapy and PAC therapy in all eligible trials in intention-to-treat analysis (A) and in per-protocol analysis (B) in the fixed-effects model. Abbreviations: CI, confidence interval; PAC, proton pump inhibitor/amoxicillin/clarithromycin; PAM, proton pump inhibitor/amoxicillin/metronidazole.

A. Area with MNZ-R/CAM-S (intention-to-treat analysis)

Study or Subgroup	PAM Events	PAM Total	PAC Events	PAC Total	Weight	Risk Ratio M-H, Fixed, 95% CI	Year
Labenz 1996	5	33	1	31	0.6%	4.70 [0.58, 37.99]	1996
Pieramico 1997	12	61	8	58	4.7%	1.43 [0.63, 3.24]	1997
Pilotto (1) 1999	8	38	6	40	3.3%	1.40 [0.54, 3.67]	1999
Pilotto (2) 1999	10	50	9	50	5.1%	1.11 [0.49, 2.50]	1999
Savarino 1999	18	80	34	80	19.3%	0.53 [0.33, 0.86]	1999
Malfertheiner 2003	67	167	56	169	31.7%	1.21 [0.91, 1.61]	2003
Bhatia 2004	48	70	41	76	22.4%	1.27 [0.98, 1.65]	2004
Koivisto 2005	23	106	10	110	5.6%	2.39 [1.19, 4.77]	2005
Loghmari 2012	20	39	14	46	7.3%	1.68 [0.99, 2.87]	2012
**Total (95% CI)**		**644**		**660**	**100.0%**	**1.22 [1.04, 1.44]**	
Total events	211		179				

Heterogeneity: Chi² = 18.61, df = 8 (P = 0.02); I² = 57%
Test for overall effect: Z = 2.50 (P = 0.01)

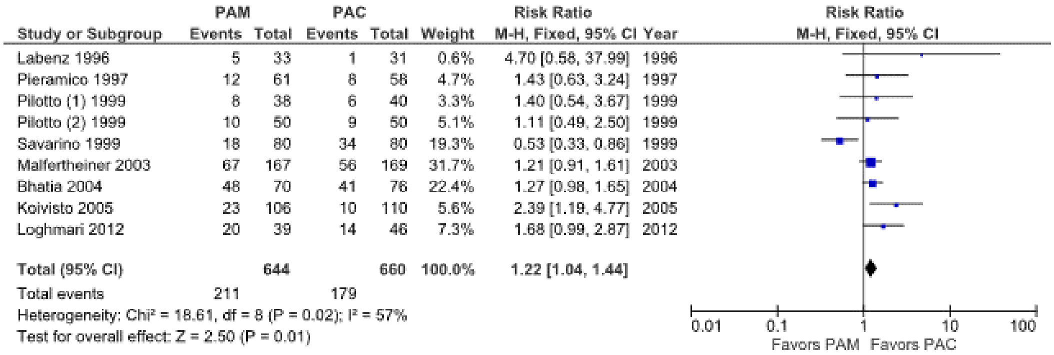

B. Area with MNZ-S/CAM-R (intention-to-treat analysis)

Study or Subgroup	PAM Events	PAM Total	PAC Events	PAC Total	Weight	Risk Ratio M-H, Fixed, 95% CI	Year
Miyaji 1997	10	70	15	74	13.9%	0.70 [0.34, 1.46]	1997
Nishizawa 2015	6	64	16	60	15.8%	0.35 [0.15, 0.84]	2015
Adachi 2017	6	72	20	68	19.6%	0.28 [0.12, 0.66]	2017
Mabe 2018	6	169	48	137	50.6%	0.10 [0.04, 0.23]	2018
**Total (95% CI)**		**375**		**339**	**100.0%**	**0.26 [0.18, 0.38]**	
Total events	28		99				

Heterogeneity: Chi² = 12.74, df = 3 (P = 0.005); I² = 76%
Test for overall effect: Z = 6.79 (P < 0.00001)

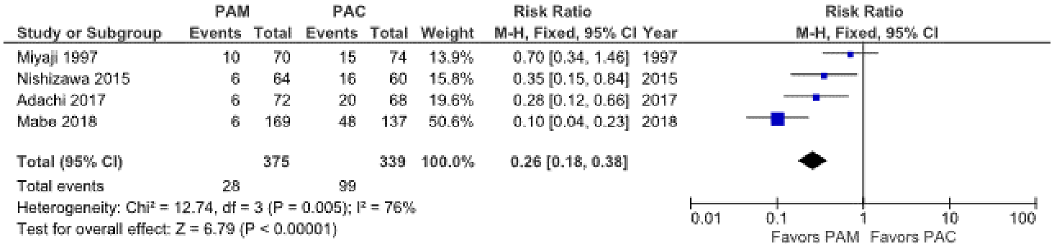

C. Area with MNZ-R/CAM-R (intention-to-treat analysis)

Study or Subgroup	PAM Events	PAM Total	PAC Events	PAC Total	Weight	Risk Ratio M-H, Fixed, 95% CI	Year
Misiewicz 1997	44	131	17	121	6.8%	2.39 [1.45, 3.95]	1997
Stack 1998	2	19	1	19	0.4%	2.00 [0.20, 20.24]	1998
Harris 1998	23	64	9	62	3.5%	2.48 [1.25, 4.92]	1998
Uygun 1999	57	134	35	88	16.3%	1.07 [0.77, 1.48]	1999
Huang 2000	9	30	4	37	1.4%	2.77 [0.95, 8.13]	2000
Namiot 2004	14	45	14	45	5.4%	1.00 [0.54, 1.85]	2004
Sun 2005	8	45	8	58	2.7%	1.29 [0.52, 3.17]	2005
Sancar 2006	5	10	4	24	0.9%	3.00 [1.01, 8.91]	2006
Namiot 2008	30	82	19	84	7.3%	1.62 [0.99, 2.63]	2008
Lahbabi 2013	31	104	25	115	9.2%	1.37 [0.87, 2.16]	2013
Lee 2015	53	170	61	170	23.6%	0.87 [0.64, 1.17]	2015
Gungor 2015	48	100	58	100	22.4%	0.83 [0.64, 1.08]	2015
**Total (95% CI)**		**934**		**923**	**100.0%**	**1.22 [1.07, 1.40]**	
Total events	324		255				

Heterogeneity: Chi² = 31.90, df = 11 (P = 0.0008); I² = 66%
Test for overall effect: Z = 2.94 (P = 0.003)

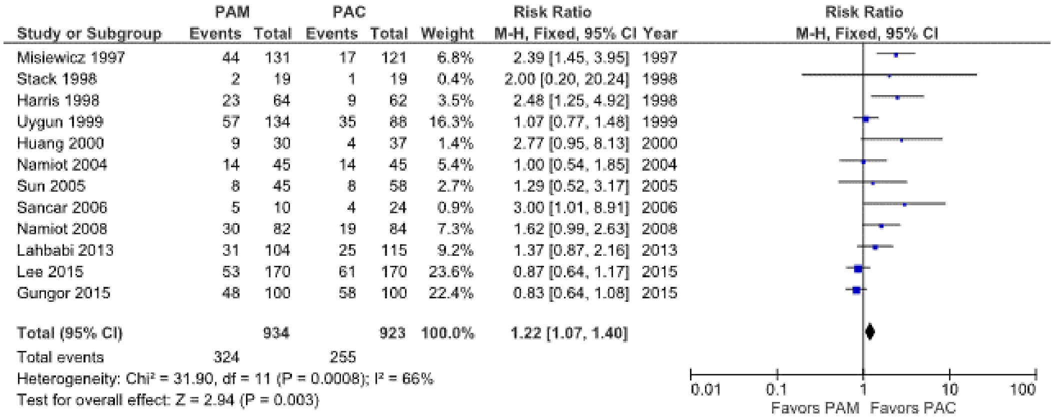

Figure S2. (**A**) Forest plots of eradication rates between PAM therapy and PAC therapy in areas classified as having low CAM resistance (< 15%) and high MNZ resistance (≥ 15%), (**B**) high CAM resistance and low MNZ resistance, and (**C**) high CAM resistance and high MNZ resistance in intention-to-treat analysis in the fixed-effects model. Abbreviations: CAM, clarithromycin; CI, confidence interval; MNZ, metronidazole; PAC, proton pump inhibitor/amoxicillin/clarithromycin; PAM, proton pump inhibitor/amoxicillin/metronidazole.

Table S1. Classification of countries based on resistance rates to CAM and MNZ.

Country	Resistance rate to CAM (95% CI)	Resistance rate to MNZ (95% CI)
Areas with high resistance rates to both CAM (≥ 15%) and MNZ (≥ 15%)		
UK	36% (30%–36%) [1]	57% (51%–63%) [1]
Turkey	28% (19%–36%) [2]	35% (26%–45%) [2]
Taiwan	26% (22%–31%) [3-6]	31% (27%–35%) [3-6]
China	37% (30%–43%) [7-11]	77% (74%–79%) [7-11]
Poland	46% (38%–53%) [12]	38% (24%-51%) [12]
Morocco	25% (33%–48%) [13]	40% (19%-33%) [13]
Korea	18% (10%–25%) [14-16]	40% (3%-50%) [14-16]
Areas with low resistance rates to CAM (< 15%) and high resistance rates to MNZ (≥ 15%)		
Germany	13% (6%–20%) [17,18]	37% (30%-43%) [17,18]
Italy	15% (11%–20%) [19]	26% (22%-31%) [19]
North Indian	12% (5%–22%) [20,21]	68% (55%-72%) [20,21]
Finland	2% (1%–4%) [22]	37% (31%-43%) [22]
Tunisia	14% (10%–20%) [23]	57% (50%-64%) [23]
Area with high resistance rates of CAM (≥ 15%) and low resistance rates to MNZ (< 15%)		
Japan	28% (25%–32%) [24–26]	6% (5%-8%) [24-27]

Abbreviations: CAM, clarithromycin; CI, confidence interval; MNZ, metronidazole; UK, United Kingdom.

Reference

1. McNulty, C.A.; Lasseter, G.; Shaw, I.; Nichols, T.; D'Arcy, S.; Lawson, A.J.; Glocker, E. Is Helicobacter pylori antibiotic resistance surveillance needed and how can it be delivered? *Alimentary pharmacology & therapeutics* 2012, *35*, 1221-1230, doi:10.1111/j.1365-2036.2012.05083.x.
2. Kocazeybek, B.; Tokman, H.B. Prevalence of Primary Antimicrobial Resistance of H. pylori in Turkey: A Systematic Review. *Helicobacter* 2016, *21*, 251-260, doi:10.1111/hel.12272.
3. Cheng, A.; Sheng, W.H.; Liou, J.M.; Wang, H.P.; Wu, M.S.; Lin, J.T.; Chang, S.C. Comparative in vitro antimicrobial susceptibility and synergistic activity of antimicrobial combinations against Helicobacter pylori isolates in Taiwan. *Journal of microbiology, immunology, and infection = Wei mian yu gan ran za zhi* 2015, *48*, 72-79, doi:10.1016/j.jmii.2012.08.021.
4. Chen, K.Y.; Lin, T.J.; Lin, C.L.; Lee, H.C.; Wang, C.K.; Wu, D.C. Hybrid vs sequential therapy for eradication of Helicobacter pylori in Taiwan: A prospective randomized trial. *World journal of gastroenterology* 2015, *21*, 10435-10442, doi:10.3748/wjg.v21.i36.10435.
5. Liou, J.M.; Chang, C.Y.; Chen, M.J.; Chen, C.C.; Fang, Y.J.; Lee, J.Y.; Wu, J.Y.; Luo, J.C.; Liou, T.C.; Chang, W.H., et al. The Primary Resistance of Helicobacter pylori in Taiwan after the National Policy to Restrict Antibiotic Consumption and Its Relation to Virulence Factors-A Nationwide Study. *PloS one* 2015, *10*, e0124199, doi:10.1371/journal.pone.0124199.
6. Wu, I.T.; Chuah, S.K.; Lee, C.H.; Liang, C.M.; Lu, L.S.; Kuo, Y.H.; Yen, Y.H.; Hu, M.L.; Chou, Y.P.; Yang, S.C., et al. Five-year sequential changes in secondary antibiotic resistance of Helicobacter pylori in Taiwan. *World journal of gastroenterology* 2015, *21*, 10669-10674, doi:10.3748/wjg.v21.i37.10669.
7. Han, R.; Lu, H.; Jiang, M.W.; Tan, K.W.; Peng, Z.; Hu, J.L.; Fang, D.C.; Lan, C.H.; Wu, X.L. Multicenter Study of Antibiotic Resistance Profile of H. pylori and Distribution of CYP2C19 Gene Polymorphism in Rural Population of Chongqing, China. *Gastroenterology research and practice* 2016, *2016*, 8547686, doi:10.1155/2016/8547686.
8. Ji, Z.; Han, F.; Meng, F.; Tu, M.; Yang, N.; Zhang, J. The Association of Age and Antibiotic Resistance of Helicobacter Pylori: A Study in Jiaxing City, Zhejiang Province, China. *Medicine* 2016, *95*, e2831, doi:10.1097/md.0000000000002831.
9. Gao, W.; Cheng, H.; Hu, F.; Li, J.; Wang, L.; Yang, G.; Xu, L.; Zheng, X. The evolution of Helicobacter pylori antibiotics resistance over 10 years in Beijing, China. *Helicobacter* 2010, *15*, 460-466, doi:10.1111/j.1523-5378.2010.00788.x.
10. Zhou, Y.Q.; Xu, L.; Wang, B.F.; Fan, X.M.; Wu, J.Y.; Wang, C.Y.; Guo, C.Y.; Xu, X.F. Modified Sequential Therapy Regimen versus Conventional Triple Therapy for Helicobacter Pylori Eradication in Duodenal Ulcer Patients in China: A Multicenter Clinical Comparative Study. *Gastroenterology research and practice* 2012, *2012*, 405425, doi:10.1155/2012/405425.
11. Su, P.; Li, Y.; Li, H.; Zhang, J.; Lin, L.; Wang, Q.; Guo, F.; Ji, Z.; Mao, J.; Tang, W., et al. Antibiotic resistance of Helicobacter pylori isolated in the Southeast Coastal Region of China. *Helicobacter* 2013, *18*, 274-279, doi:10.1111/hel.12046.
12. Binkowska, A.; Biernat, M.M.; Laczmanski, L.; Gosciniak, G. Molecular Patterns of Resistance Among Helicobacter pylori Strains in South-Western Poland. *Frontiers in microbiology* 2018, *9*, 3154, doi:10.3389/fmicb.2018.03154.
13. Bouihat, N.; Burucoa, C.; Benkirane, A.; Seddik, H.; Sentissi, S.; Al Bouzidi, A.; Elouennas, M.; Benouda, A. Helicobacter pylori Primary Antibiotic Resistance in 2015 in Morocco: A Phenotypic and Genotypic Prospective and Multicenter Study. *Microbial drug resistance (Larchmont, N.Y.)* 2017, *23*, 727-732, doi:10.1089/mdr.2016.0264.
14. Lee, J.W.; Kim, N.; Kim, J.M.; Nam, R.H.; Chang, H.; Kim, J.Y.; Shin, C.M.; Park, Y.S.; Lee, D.H.; Jung, H.C. Prevalence of primary and secondary antimicrobial resistance of Helicobacter pylori in Korea from 2003 through 2012. *Helicobacter* 2013, *18*, 206-214, doi:10.1111/hel.12031.
15. Chung, J.W.; Lee, G.H.; Jeong, J.Y.; Lee, S.M.; Jung, J.H.; Choi, K.D.; Song, H.J.; Jung, H.Y.; Kim, J.H. Resistance of Helicobacter pylori strains to antibiotics in Korea with a focus on fluoroquinolone resistance. *Journal of gastroenterology and hepatology* 2012, *27*, 493-497, doi:10.1111/j.1440-1746.2011.06874.x.
16. Hwang, T.J.; Kim, N.; Kim, H.B.; Lee, B.H.; Nam, R.H.; Park, J.H.; Lee, M.K.; Park, Y.S.; Lee, D.H.; Jung, H.C., et al. Change in antibiotic resistance of Helicobacter pylori strains and the effect of A2143G point mutation of 23S rRNA on the eradication of H. pylori in a single center of Korea. *Journal of clinical gastroenterology* 2010, *44*, 536-543, doi:10.1097/MCG.0b013e3181d04592.
17. Wueppenhorst, N.; Stueger, H.P.; Kist, M.; Glocker, E. Identification and molecular characterization of triple- and quadruple-resistant Helicobacter pylori clinical isolates in Germany. *The Journal of antimicrobial chemotherapy* 2009, *63*, 648-653, doi:10.1093/jac/dkp003.

18. Wuppenhorst, N.; Draeger, S.; Stuger, H.P.; Hobmaier, B.; Vorreiter, J.; Kist, M.; Glocker, E.O. Prospective multicentre study on antimicrobial resistance of Helicobacter pylori in Germany. *The Journal of antimicrobial chemotherapy* 2014, *69*, 3127-3133, doi:10.1093/jac/dku243.
19. De Francesco, V.; Giorgio, F.; Ierardi, E.; Zotti, M.; Neri, M.; Milano, A.; Varasano, V.; Luzza, F.; Suraci, E.; Marmo, R., et al. Primary clarithromycin resistance in Helicobacter pylori: the Multicentric Italian Clarithromycin Resistance Observational (MICRO) study. *Journal of gastrointestinal and liver diseases : JGLD* 2011, *20*, 235-239.
20. Wani, F.A.; Bashir, G.; Khan, M.A.; Zargar, S.A.; Rasool, Z.; Qadri, Q. Antibiotic resistance in Helicobacter pylori: A mutational analysis from a tertiary care hospital in Kashmir, India. *Indian journal of medical microbiology* 2018, *36*, 265-272, doi:10.4103/ijmm.IJMM_18_19.
21. Gehlot, V.; Mahant, S.; Mukhopadhyay, A.K.; Das, K.; De, R.; Kar, P.; Das, R. Antimicrobial susceptibility profiles of Helicobacter pylori isolated from patients in North India. *Journal of global antimicrobial resistance* 2016, *5*, 51-56, doi:10.1016/j.jgar.2015.09.009.
22. Koivisto, T.T.; Rautelin, H.I.; Voutilainen, M.E.; Heikkinen, M.T.; Koskenpato, J.P.; Farkkila, M.A. First-line eradication therapy for Helicobacter pylori in primary health care based on antibiotic resistance: results of three eradication regimens. *Alimentary pharmacology & therapeutics* 2005, *21*, 773-782, doi:10.1111/j.1365-2036.2005.02398.x.
23. Ben Mansour, K.; Burucoa, C.; Zribi, M.; Masmoudi, A.; Karoui, S.; Kallel, L.; Chouaib, S.; Matri, S.; Fekih, M.; Zarrouk, S., et al. Primary resistance to clarithromycin, metronidazole and amoxicillin of Helicobacter pylori isolated from Tunisian patients with peptic ulcers and gastritis: a prospective multicentre study. *Annals of clinical microbiology and antimicrobials* 2010, *9*, 22, doi:10.1186/1476-0711-9-22.
24. Murakami, K.; Furuta, T.; Ando, T.; Nakajima, T.; Inui, Y.; Oshima, T.; Tomita, T.; Mabe, K.; Sasaki, M.; Suganuma, T., et al. Multi-center randomized controlled study to establish the standard third-line regimen for Helicobacter pylori eradication in Japan. *Journal of gastroenterology* 2013, *48*, 1128-1135, doi:10.1007/s00535-012-0731-8.
25. Horiki, N.; Omata, F.; Uemura, M.; Suzuki, S.; Ishii, N.; Iizuka, Y.; Fukuda, K.; Fujita, Y.; Katsurahara, M.; Ito, T., et al. Annual change of primary resistance to clarithromycin among Helicobacter pylori isolates from 1996 through 2008 in Japan. *Helicobacter* 2009, *14*, 86-90, doi:10.1111/j.1523-5378.2009.00714.x.
26. Asaka, M.; Kato, M.; Takahashi, S.; Fukuda, Y.; Sugiyama, T.; Ota, H.; Uemura, N.; Murakami, K.; Satoh, K.; Sugano, K. Guidelines for the management of Helicobacter pylori infection in Japan: 2009 revised edition. *Helicobacter* 2010, *15*, 1-20, doi:10.1111/j.1523-5378.2009.00738.x.
27. Kobayashi, I.; Murakami, K.; Kato, M.; Kato, S.; Azuma, T.; Takahashi, S.; Uemura, N.; Katsuyama, T.; Fukuda, Y.; Haruma, K., et al. Changing antimicrobial susceptibility epidemiology of Helicobacter pylori strains in Japan between 2002 and 2005. *Journal of clinical microbiology* 2007, *45*, 4006-4010, doi:10.1128/jcm.00740-07.
